# A comparative analysis of the cost-utility of the Philippine tax on sweetened beverages as proposed and as implemented

**DOI:** 10.1016/j.lanwpc.2023.100912

**Published:** 2023-09-27

**Authors:** Oliver Huse, Kathryn Backholer, Phuong Nguyen, Anthony Calibo, Mildred Guirindola, Josie P. Desnacido, Gary Sacks, Andrew Colin Bell, Anna Peeters, Imelda Angeles-Agdeppa, Jaithri Ananthapavan

**Affiliations:** a Global Centre for Preventative Health and Nutrition, Institute for Health Transformation, Faculty of Health, Deakin University, Geelong, Australia; b Deakin Health Economics, Institute for Health Transformation, Faculty of Health, Deakin University, Geelong, Australia; c Child Health Division, Department of Health, Medical Specialist IV, Disease Prevention and Control Bureau, Manila (2011-2020), Philippines; d Institute of Pediatrics and Child Health, St. Luke's Medical Center, Quezon City, Philippines; e Department of Science and Technology, Food and Nutrition Research Institute, Manila, Philippines

**Keywords:** Cost-utility, Sugar sweetened beverages, Taxation, Public health policy, Obesity prevention, Philippines, Corporate political activity

## Abstract

**Background:**

In response to increasing overweight and obesity, the Philippine government introduced a tax on sweetened beverages (SBs) in 2018. Evidence suggests that the beverage industry influenced the final tax design, making it more favourable for industry than the initially proposed bill. This study aimed to compare the relative health and economic benefits of the proposed SB tax with the implemented SB tax.

**Methods:**

Philippine dietary consumption data were combined with price elasticity data from Mexico and data from Australia adapted to the Philippine context to estimate reductions in SB purchases and changes in body mass index (BMI) following the implementation of the tax. A multi-state, multiple-cohort Markov model was used to estimate the change in health-adjusted life years (HALYs) due to reduction in the epidemiology of obesity-related diseases, healthcare cost savings and government taxation revenue, resulting from both the proposed and implemented tax policies, over the lifetime of the 2018 Philippine population.

**Findings:**

The proposed and implemented taxes were modelled to be dominant (cost-saving and improving health). Intervention costs were modelled to be PHP305.2 million (M) (approximately US$6M). Compared to the proposed tax, the implemented tax was modelled to result in a 43.0% smaller reduction in targeted beverage intake (51.1 ml/person/day vs. 89.7 ml/person/day), a 43.5% smaller reduction in BMI (0.35 kg/m^2^ vs. 0.62 kg/m^2^), 39.7% fewer HALYs gained (2,503,118 vs. 4,149,030), 39.9% fewer healthcare cost savings (PHP16.4 billion (B) vs. PHP27.3B), and 27.7% less government taxation revenue (PHP426.3B vs. PHP589.4B).

**Interpretation:**

While the implemented tax in the Philippines will benefit population health, it is likely to yield less benefit than the proposed tax. The influence of the food and beverage industry on policy processes has the potential to lessen the benefits of population NCD prevention policies.

**Funding:**

OH was supported to conduct this research by an Australian Government Research Training Program Stipend Scholarship. The funding body had no role in data collection and analysis, or manuscript preparation.

**Research in context**

**Evidence before this study**

In 2018, the Philippine Government introduced a tax on sweetened beverages to combat rising rates of overweight and obesity. However, a study by Huse et al. (2023) documents the pervasive influence of the unhealthy food and beverage industry in this country. Onagan et al. (2018) describe how lobbying from the beverage industry influenced the final tax design, making it more favourable for industry than the initially proposed bill. Changes to the policy design, likely in response to industry interference, included a lower tax rate and the exclusion of some products from taxation. While the Philippine sweetened beverage tax has been modelled before (Saxena et al.), this study aimed to build on this prior research and quantify the influence of the food and beverage industry over policy by comparing the relative health and economic benefits of the proposed sweetened beverage tax with the currently implemented Philippine sweetened beverage tax.

**Added value of this study**

We find that the currently implemented Philippine sweetened beverage tax is likely to be highly cost effective and resulting in substantial health-care cost-savings and government revenue. However, compared to the proposed tax, the implemented tax was modelled to result in a 43% smaller reduction in targeted beverage intake, a 44% smaller reduction in BMI, 40% fewer long term health gains (quantified as health-adjusted life years), 40% fewer healthcare cost savings, and 28% less government taxation revenue. It is likely that corporate lobbying by the beverage industry is responsible for at least part of the missed potential benefits from this policy.

**Implications of all the available evidence**

Our study provides support for the continuation and expansion of the Philippine sweetened beverage tax. However, it also provides evidence of the need for strong conflicts of interest and transparency policies, in the Philippines and other lower-middle income countries in the Western Pacific region.

## Introduction

Countries in East Asia and the Pacific are facing an increased burden of diet-related noncommunicable diseases (NCDs).^1^, ^2^, ^3^, ^4^, ^5^, ^6^ This is being fuelled by a shift in diets away from traditional foods, meal preparations, and cuisines towards greater consumption of animal-sourced and ultra-processed foods (UPFs).^3^^,^^5^^,^^7^, ^8^, ^9^, ^10^ Sweetened beverages (SBs), which are beverages sweetened with either artificial or caloric sweeteners, are one key UPF that have been linked with significant health consequences, and consumption of these beverages is known to be increasing in many East Asian and Pacific Island countries.^3^^,^^5^^,^^7^, ^8^, ^9^, ^10^ In response to the increasing prevalence of overweight and obesity,^11^ the Philippine national government introduced a tax on SBs in 2018 as part of a broader tax reform (Republic Act 10963 Section 47),^12^ and preliminary evidence suggests that this policy could improve population health by reducing energy and sugar intakes and subsequently reducing the risk of overweight, obesity, and related health conditions.^13^ As at September 2023, the SB tax remains in place. The SBs included under the tax were sweetened juice drinks, sweetened teas, carbonated beverages, flavoured waters, energy and sports drinks, powdered drinks, cereal and grain-based beverages, and other beverages containing added sugar.^12^^,^^13^ Studies from Chile^14^ and Mexico^15^, ^16^, ^17^, ^18^ demonstrate that taxation of these beverages can increase the price of targeted products and subsequently reduce purchasing and consumption. Economic modelling studies conducted in Australia,^19^ India,^20^ Mexico,^21^ South Africa,^22^ Thailand,^23^ and the United Kingdom^24^ have highlighted the potential for SB taxation policies to reduce disease burden and improve population health in the long-term, whilst also resulting in reduced healthcare costs and increased government revenue.

Evidence shows that the UPF industry works to influence government policies in ways that support corporate revenues and profits, and protect against regulatory threats, often at the expense of public health.^25^ The market and political power of transnational food corporations in a globalized economy continue to increase,^26^^,^^27^ accompanied by concerns about their undue influence over food and nutrition governance and policy processes.^28^, ^29^, ^30^, ^31^, ^32^, ^33^ Corporate political activities (CPA) refers to attempts by corporations to influence government actions, with evidence that UPF companies seek to defeat, delay, weaken, circumvent, and/or overturn proposed and implemented food and nutrition policies.^34^ To influence these policies, the UPF industry has been observed to use a wide range of strategies adopted across various countries,^28^^,^^30^, ^31^, ^32^, ^33^ even in small island states like Fiji.^29^ CPA has been shown to be effective in limiting the scope of nutrition policies^28^, ^29^, ^30^, ^31^ and, in some cases, preventing their introduction all together.^35^^,^^36^

In the case of the Philippines, there is evidence that food industry CPA, and their lobbying activities in particular, influenced the final version of the 2018 SB tax design, making it more favourable for the beverage industry (and less effective from a public health perspective) than the initially proposed SB tax (House Bill 292, 17th Congress).^12^^,^^37^ Specific changes attributed to industry influence included reductions in the tax rate (for beverages sweetened by caloric and non-caloric sweeteners other than high fructose corn syrup) and the exclusion of sweetened coffee-based beverages. Coffee-based beverages are the second most consumed beverage in the Philippines behind water,^38^ and 3-in-1 instant mixes (coffee, sugar and cream powder) are particularly popular.^39^ Coffee-based beverages are also consumed amongst children and adolescents in the Philippines.^40^

The health and economic impacts of the Philippine SB tax have been modelled previously^13^^,^^41^ and these modelling studies found reductions in carbonated beverage consumption, disease deaths, and healthcare costs, and increased government revenue, in response to the policy. However, there were several limitations of these studies that the current study will improve: i) Saxena et al.^13^ did not use the most up-to-date national survey on SB consumption, conducted in the same year that the tax was implemented, ii) cross-price elasticities to estimate the likely impact of substitution to alternative beverages following the implementation of the tax were not included, iii) healthcare savings were based solely on reduced hospitalizations, iv) the costs of policy implementation and monitoring were not included, and v) colon, breast, endometrial, and kidney cancer, hypertensive heart disease, and hip and knee osteoarthritis were not included (Saxena et al.^13^ included type 2 diabetes; ischaemic heart disease, and; stroke).

The aim of this study was to compare the relative health and economic benefits of the proposed SB tax with the currently implemented Philippine SB tax. The goal was to estimate the potential reductions in health and economic benefits resulting from CPA aimed at weakening/delaying the SB tax policy.

## Methods

Cost-utility analyses can be used to assess the relative costs and benefits of various policy options and policy formulations.^42^

### Study design and modelling approach

This was a modelled economic evaluation estimating the costs and benefits arising from the currently implemented Philippine SB tax compared to what could have arisen from the initially proposed SB tax. We took a whole of government perspective in the analysis and estimated the cost of policy implementation, taxation revenue, potential healthcare cost savings and health benefits. For the primary analysis, two scenarios were run, one to estimate the benefits from the proposed SB tax and one to estimate the benefits of the implemented SB tax. The intervention was modelled over the lifetime of the 2018 Philippines population (the modelled population was a closed cohort). Evidence from Mexico^17^ and Oakland^43^ suggests that behavioural changes in response to SB taxation policies are sustained.

### Intervention specification

Table 1 describes the characteristics of the proposed SB tax and the currently implemented SB tax, including the definition of SBs, products included, the taxation rate and notable exclusions for each iteration of the policy.^12^ While the SB tax underwent several iterations through the policy development process, Huse et al.^37^ have suggested that CPA is conducted by the food and beverage industry at all stages of policy development and implementation processes in the Philippines, and so this study compares the initially proposed SB tax with the currently implemented SB tax. Notable changes between the initially proposed SB tax and the currently implemented SB tax were the exclusion of coffee-based beverages and a lower tax rate for beverages sweetened with caloric or non-caloric sweeteners other than high fructose corn syrup. While the implemented SB tax included a higher rate for beverages sweetened with high fructose corn syrup, we assumed that manufacturers would shift to other caloric sweeteners in order to receive a lower tax rate, and so modelled the tax rate at 6 Philippine pesos (PHP) per litre. Both SB tax scenarios were compared to a do-nothing scenario, where the Philippine government had not implemented any SB tax.

**Table 1:** Characteristics of the proposed Philippine SB tax and the implemented Philippine SB tax.

	Proposed tax (House Bill 292)	Implemented tax (Republic Act 10963 Section 47)
Definition of sweetened beverages	Non-alcoholic beverages that contain caloric sweeteners or added sugar or artificial or non-caloric sweeteners in the form of a liquid, syrup, concentrate or solid mixture that is added to water or other liquids to make a drink	Non-alcoholic beverages of any constitution that are pre-packaged and sealed in accordance with Philippine Food and Drug Administration (FDA) standards and that contain caloric or non-caloric sweeteners or both added by the manufacturers
Included products^a^	Soft drinks and carbonated drinks; Fruit drinks and punches; Sports and energy drinks; Sweetened tea and coffee-based products; All non-alcoholic beverages (ready to drink (RTD) or powder form) that contain added natural or artificial sugar	All carbonated beverages; Sweetened juice drinks; Sports and energy drinks; Sweetened tea; Flavoured water; Powdered drinks not classified as milk, juice or tea; Cereal and grain-based beverages; Other non-alcoholic beverages that contain added sugar
Taxation rate	10 PHP per litre	6 PHP per litre for beverages sweetened with caloric or non-caloric sweeteners other than high fructose corn syrup 12 PHP per litre for beverages sweetened with high fructose corn syrup
Notable exclusions	All milk and yoghurt-based products; 100% natural fruit and vegetable juices; Meal-replacement beverages	All milk-based products; 100% natural fruit and vegetable juices; Meal-replacement beverages; coffee-based products; Beverages sweetened with coconut sap or stevia glycosides

a As defined by the Philippine 2018–2019 Expanded National Nutrition Survey (ENNS) Food Consumption Survey.^44^

### Health impact modelling

#### Effect of the tax on SB purchases, SB consumption and energy intake

**Fig. 1:**
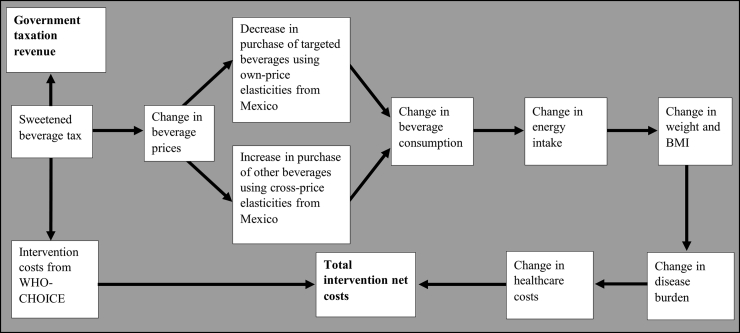
**Logic pathway for modelling the health impacts of the Philippine SB tax**.

Fig. 1 depicts the logic pathway that demonstrates how the Philippine SB tax results in a reduction in beverage consumption, improved health outcomes, changes to government revenue and healthcare savings. As it is producers who pay the SB tax in the Philippines, it was hypothesised that the tax was completely passed through to consumers, resulting in an increased price of taxed beverages. While there is no available data supporting this for the Philippines, a 100% pass-through rate has been observed internationally,^45^ and modelled in the Philippine context.^13^ Using price and cross-price elasticities, the increase in the price of taxed beverages was used to estimate the change in purchasing of taxed, and complementary and substitute products. The net change in sugar consumption and consequent impact on energy intake was used to estimate changes in body weight, BMI, and subsequent health outcomes for the modelled population. The model also accounted for substitution from taxed beverages to non-taxed beverages. A broad range of tax reforms were implemented in the Philippines at the time of the SB tax, meaning that any potential price elasticities may not accurately assess the impact on consumption. As such, we used price elasticities from Mexico, another low-middle income country with reliable price elasticity estimates available.^46^ This was used previously to estimate the impact of a SB tax in the Philippines.^13^ While Mexico and the Philippines differ in some respects (GDP per capita, pre-tax SB consumption, urban-rural population distribution), they are comparable in others (life expectancy at birth, population size and age distribution, Gini coefficient) (Supplementary File S1). We did not consider the potential impact of product reformulation by industry in response to the SB tax due a to a lack of available data.

To estimate the change in mean energy intake as a result of the SB tax, we first converted the Philippine SB tax rate to a percentage, based on the mean price of included SBs in the Philippines as calculated by Saxena et al.^13^: an effective 13.3% price increase for the implemented SB tax, and a 22.2% price increase for the initially proposed SB tax. We then multiplied this percentage change in price with price elasticities to obtain the percentage change in quantity of included beverages consumed under each tax scenario. This was used to estimate the change in quantity of included beverages consumed using beverage consumption data from the Philippine 2018–2019 Expanded National Nutrition Survey (ENNS) Food Consumption Survey.^44^ Beverage consumption data was available for men and women (combined) aged ≤5 years, 6–12 years, 13–18 years, 19–59 years, and ≥60 years. Given the lack of data on the nutrient composition of beverages available for sale in the Philippines, Australian food composition data was used. The Australian Food Composition Database states that soft drinks sweetened with calorific sweeteners have a mean energy density of 174 kilojoules (kJ)/100 ml, sports drinks and energy drinks have an energy density of 191kJ100 mL juices and juice drinks have an energy density of 184 kJ/100 ml, milk- and grain-based beverages have an energy density of 266 kJ/100 ml, and coffee-based beverages have an energy density of 260 kJ/100 mL.^47^ These values are comparable to those used in Indonesia^48^ (however this source provided the energy density of fewer products).

#### Effect of the tax on body weight and health outcomes

Validated energy balance equations were used to calculate changes in body weight for each age and sex group as a result of this estimated reduction in mean daily energy intake.^49^^,^^50^ Changes in weight were converted to changes in body mass index (BMI) using the Philippine 2018 ENNS data on average height and weight of each age and sex group.^44^ The effect of the tax on consumption was assumed to last the lifetime of the modelled population.

The previously developed and validated ACE-Obesity Policy model,^51^ which has previously been used in other taxation modelling studies,^19^^,^^52^^,^^53^ was adapted to the Philippine context and used to estimate the long-term health outcomes resulting from a change in population BMI. The model is a proportional, multi-state life table Markov model. All outcomes were modelled for the 2018 Philippine population aged 2–100 years.^44^ 2018 was selected as the reference year to align with the year that the tax was implemented. Details of the ACE-Obesity Policy model have been previously published^54^ and are described here briefly. The ACE-Obesity Policy model estimates the change in the epidemiology of nine obesity-related diseases (type 2 diabetes; hypertensive heart disease; ischaemic heart disease; stroke; osteoarthritis of the hip and knee; kidney cancer; colorectal cancer; endometrial cancer, and; breast cancer) resulting from a given policy compared to the counterfactual (no policy). Modelling the epidemiology of each of the diseases for the Philippines required data on incidence, prevalence and case fatality rates. Data were sourced from the Global Burden of Disease study and transitions between the four health states for each of the diseases (healthy, diseased, dead due to disease, and dead from other causes) were calculated using the DisMod II software package.^55^ The long-term health outcome was the incremental Health-Adjusted Life Years (HALYs) gained. HALYs were calculated by aggregating the population level changes to overall mortality and morbidity for each disease (using Global Burden of Disease disability weights,^56^ and utility weights calculated using the EQ-5D to quantify the quality of life impact of overweight and obesity in childhood^57^^,^^58^).

### Cost modelling

#### Taxation implementation costs

The World Health Organization-Choosing Interventions that are Cost-Effective (WHO-CHOICE) is a cost-effectiveness study that takes a standardized approach to estimating policy costs and benefits for a range of policies across regions.^59^ The WHO-CHOICE approach has been adopted for estimating the costs associated with implementing tax increases on tobacco products in Southeast Asia.^60^ These costs were estimated to be US$ 5,400,016 per 10 million population in 2010, for the lifetime of the modelled population, and included human resource costs, consultation costs, training costs, and policy monitoring and evaluation costs.^60^ The WHO-CHOICE model has previously been used by the Philippine Department of Health (DOH) to estimate the costs of implementing a range of interventions, including tobacco, alcohol and salt-reduction policies.^61^ We used the estimated costs of raising taxes on tobacco as a proxy for the cost of implementing a tax on SBs in the Philippines. In this analysis the reported 2010 costs per 10 million population were first converted from US$ to PHP using World Bank 2010 exchange rates,^62^ then inflated to PHP 2018 values using World Bank data on annual inflation of consumer prices in the Philippines,^63^ and finally applied to the 2018 Philippine population size (according to Global Burden of Disease Study data).^56^

#### Healthcare cost savings

The ACE-Obesity Policy model includes 2015 and 2001 annual healthcare costs per incident cases (all cancers in the model) or prevalent cases (other diseases in the model), provided by the Australian Institute of Health and Welfare (AIHW).^64^ There is no data available to estimate the annual healthcare cost of treating a case of disease in the Philippines. Accordingly, to reflect the Philippine context, the Australian cost data included in the model was adjusted by the proportionate difference in annual healthcare expenditure that is allocated to treating cancers, cardiovascular diseases, and diabetes in Australia^65^ and the Philippines.^61^ The calculated costs per case of disease were relatively similar to the disease costs used by Saxena et al.^13^ in their modelling of the Philippine SB tax (which was limited to the cost of hospitalization for type 2 diabetes, ischaemic heart disease, and stroke).

#### Taxation revenue

We estimated taxation revenue resulting from both tax scenarios, as government reports on tax revenue derived from the implemented SB tax were not publicly available. The mean price of a litre of SBs in the Philippines in 2018, as calculated by Saxena et al.,^13^ was multiplied by the tax rate to calculate revenue per litre of SBs sold. This value was then multiplied by the total SB consumption following the implementation of both tax scenarios to calculate the total tax revenue gained from each policy. The input parameters and sources for these parameters are detailed in Supplementary File S2.

### Cost-utility modelling

The cost-utility analysis was based on the incremental costs and benefits for the modelled reference population compared to the intervention population. The intervention population was identical to the reference population, except that BMI was adjusted to reflect changes in energy intake resulting from the SB taxation scenarios. The Incremental Cost-Effectiveness Ratio (ICER) was calculated by dividing the incremental net costs of each of the tax scenarios by the incremental HALYs of each scenario compared to the no policy comparator. Net costs were the total healthcare cost savings less the net policy implementation and monitoring costs. Taxation revenue was considered a transfer rather than a policy cost or benefit and so was not included in net cost calculations. An ICER less than or equal to the 2018 per capita GDP of the Philippines (PHP168,236^66^) was considered cost-effective, to align with previous willingness-to-pay thresholds used in the Philippines.^67^^,^^68^

All future costs and HALYs were discounted at 3% to 2018 values, as recommended by a consensus panel of health economists,^69^ and to align with previous discount rates used in cost-effectiveness analyses in the Philippines.^67^^,^^68^ Incident cases of disease have been reported without being discounted. All input variables had uncertainty incorporated and means and 95% uncertainty intervals (UI) for all modelled outputs were estimated using Monte Carlo simulations (2000 repetitions) using Ersatz software (version 1.3).^70^ Input variable means and distributions are reported in Supplementary File S2.

The Consolidated Health Economic Evaluation Reporting Checklist is reported in Supplementary File S3.^71^

### Sensitivity analyses

Several plausible scenarios were tested in the sensitivity analyses to assess the impact on the cost-utility results (Table 2).

**Table 2:** Sensitivity analysis scenarios.

Scenario	Notable untaxed products	Tax rate	Other notes
Scenario A	All milk and yoghurt-based products; 100% natural fruit and vegetable juices; Meal-replacement beverages	6.00 PHP per litre	–
Scenario B	All milk-based products; 100% natural fruit and vegetable juices; Meal-replacement beverages; coffee-based products; Beverages sweetened with coconut sap or stevia glycosides	10.00 PHP per litre	–
Scenario C	100% natural fruit and vegetable juices; Meal-replacement beverages	10.00 PHP per litre	–
Scenario D	All milk-based products; 100% natural fruit and vegetable juices; Meal-replacement beverages; coffee-based products; Beverages sweetened with coconut sap or stevia glycosides	6.00 PHP per litre	Elasticities derived from Chile
Scenario E	All milk-based products; 100% natural fruit and vegetable juices; Meal-replacement beverages; coffee-based products; Beverages sweetened with coconut sap or stevia glycosides	6.00 PHP per litre	Healthcare cost per incident/prevalent case of disease halved
Scenario F	All milk-based products; 100% natural fruit and vegetable juices; Meal-replacement beverages; coffee-based products; Beverages sweetened with coconut sap or stevia glycosides	6.00 PHP per litre	Policy implementation and monitoring costs doubled
Scenario G	All milk-based products; 100% natural fruit and vegetable juices; Meal-replacement beverages; coffee-based products; Beverages sweetened with coconut sap or stevia glycosides	6.00 PHP per litre	Utility weights taken from a systematic review

First, we tested the impact of the various changes made to the proposed SB tax to account for variations in the impact of industry influence. Three additional scenarios were modelled: i) a scenario where coffee-based beverages were included (as per the proposed SB tax) but the tax rate was 6 PHP per litre (as per the implemented SB tax) (Scenario A), ii) a scenario where coffee-based beverages were excluded (as per the implemented SB tax) but the tax rate was 10 PHP per litre (as per the proposed SB tax) (Scenario B), and iii) a ‘best case’ scenario, with coffee- and milk-based beverages included (in contrast to the implemented and proposed SB taxes) and a tax rate of 10 PHP per litre (Scenario C). We expected that as the tax rate increased, and additional products were included, health and economic benefits would increase.

Second, we wished to test the impact of uncertainty surrounding the modelling data sources and assumptions. Four scenarios were modelled: i) a scenario with price elasticities drawn from a study conducted in Chile^72^ (to account for uncertainty surrounding the transferability of price elasticities from Mexico to the Philippine context) (Scenario D), ii) a scenario with the cost per case of disease reduced by 50% (given the lack of data on the cost of incident and prevalent cases of disease in the Philippines) (Scenario E), iii) a scenario with the estimated policy costs doubled (given the lack of data on the cost of implementing a SB taxation policy in the Philippines) (Scenario F), and a scenario where utility weights quantifying the quality of life impact of overweight and obesity in childhood were drawn from a systematic review^73^ (as opposed to a study conducted in Australia) (Scenario G).

### Role of the funding source

OH was supported to conduct this research by an Australian Government Research Training Program Stipend Scholarship. The funding source had no role in study design, data collection, data analysis, interpretation, or writing of this report.

## Results

### Intervention effectiveness

Table 3 shows the estimated reductions in energy intake and the corresponding decrease in weight resulting from the implemented SB tax and the proposed SB tax in the Philippines.

**Table 3:** Population change in beverage consumption, energy intake, weight, and BMI.

Parameter	Implemented SB tax	Proposed SB tax
Weighted mean change in volume of soft drinks consumed (ml per person per day)^a^	−7.0 (95% UI: −7.6 to −6.4)	−11.6 (95% UI: −12.6 to −11.6)
Weighted mean change in volume of energy and sports drinks consumed (ml per person per day)^a^	−10.0 (95% UI: −10.9 to −9.1)	−16.6 (95% UI: −18.1 to −15.1)
Weighted mean change in volume of juice consumed (ml per person per day)^a^	−9.2 (95% UI: −10.1 to −8.4)	−15.4 (95% UI: −16.8 to −14.0)
Weighted mean change in volume of sweetened tea consumed (ml per person per day)^a^	−9.7 (95% UI: −10.6 to −8.8)	−16.1 (95% UI: −17.6 to −14.7)
Weighted mean change in volume of sweetened powdered beverages consumed (ml per person per day)^a^	−4.5 (95% UI: −5.0 to −4.1)	−7.6 (95% UI: −8.3 to −6.9)
Weighted mean change in volume of cereal and grain-based beverages consumed (ml per person per day)^a^	−5.2 (95% UI: −5.6 to −4.7)	−8.7 (95% UI: −9.4 to −7.9)
Weighted mean change in volume of coffee-based beverages consumed (ml per person per day)^a^	0.2 (95% UI: 0.1–0.2)	−4.3 (95% UI: −4.7 to −3.9)
Weighted mean change in volume of milk-based beverages consumed (ml per person per day)^a^	0.1 (95% UI: 0.0–0.1)	0.1 (95% UI: 0.1–0.1)
Weighted mean change in volume of other sugary drink types consumed (ml per person per day)^a^	−5.7 (95% UI: −6.2 to −5.2)	−9.5 (95% UI: −10.4 to −8.7)
Weighted mean change in volume of all taxed and untaxed beverages consumed (ml per person per day)^a^	−51.1 (95% UI: −53.1 to −49.0)	−89.7 (95% UI: −93.2 to −86.2)
Weighted mean change in energy intake (kJ per person per day)^a^	−78.7 (95% UI: −95.1 to −62.2)	−143.8 (95% UI: −171.6 to −116.3)
Weighted mean change in weight (kg per person)^a^	−0.73 (95% UI: −0.89 to −0.58)	−1.35 (95% UI: −1.60 to −1.09)
Weighted mean change in BMI (kg/m^2^ per person)^a^	−0.35 (95% UI: −0.42 to −0.28)	−0.62 (95% UI: −0.73 to −0.50)

a Weighted by the age and sex distribution of the 2018 Philippine population; Negative values equate to decreases in volume, energy intake, weight, and BMI.

The proposed SB tax was predicted to result in a population weighted mean reduction in daily SB consumption of 89.7 ml/day (95% UI: 86.2 ml/day; 93.2 ml/day) compared to 51.1 ml/day (95% UI: 49.0 ml/day; 53.1 ml/day) for the implemented SB tax. For the proposed SB tax this translated to a reduction in weighted mean daily energy intake of 143.8 kJ/day (95% UI: 116.3 kJ/day; 171.6 kJ/day) per person, while the proposed SB tax resulted in a reduction in mean daily energy intake that was 45.3% less at 78.7 kJ/day (95% UI: 62.2 kJ/day; 95.1 kJ/day) per person.

Subsequently, the proposed SB tax was modelled to result in a population weighted mean reduction in body weight of 1.35 kg (95% UI: 1.09 kg; 1.60 kg) and BMI of 0.62 kg/m^2^ (95% UI: 0.50 kg/m^2^; 0.73 kg/m^2^). Meanwhile, the implemented SB tax was modelled to result in a population weighted mean reduction in body weight of 0.73 kg (95% UI: 0.58 kg; 0.89 kg) and BMI of 0.35 kg/m^2^ (95% UI: 0.28 kg/m^2^; 0.42 kg/m^2^); 45.9% and 43.5% smaller reductions, respectively.

### Cost-utility

**Fig. 2:**
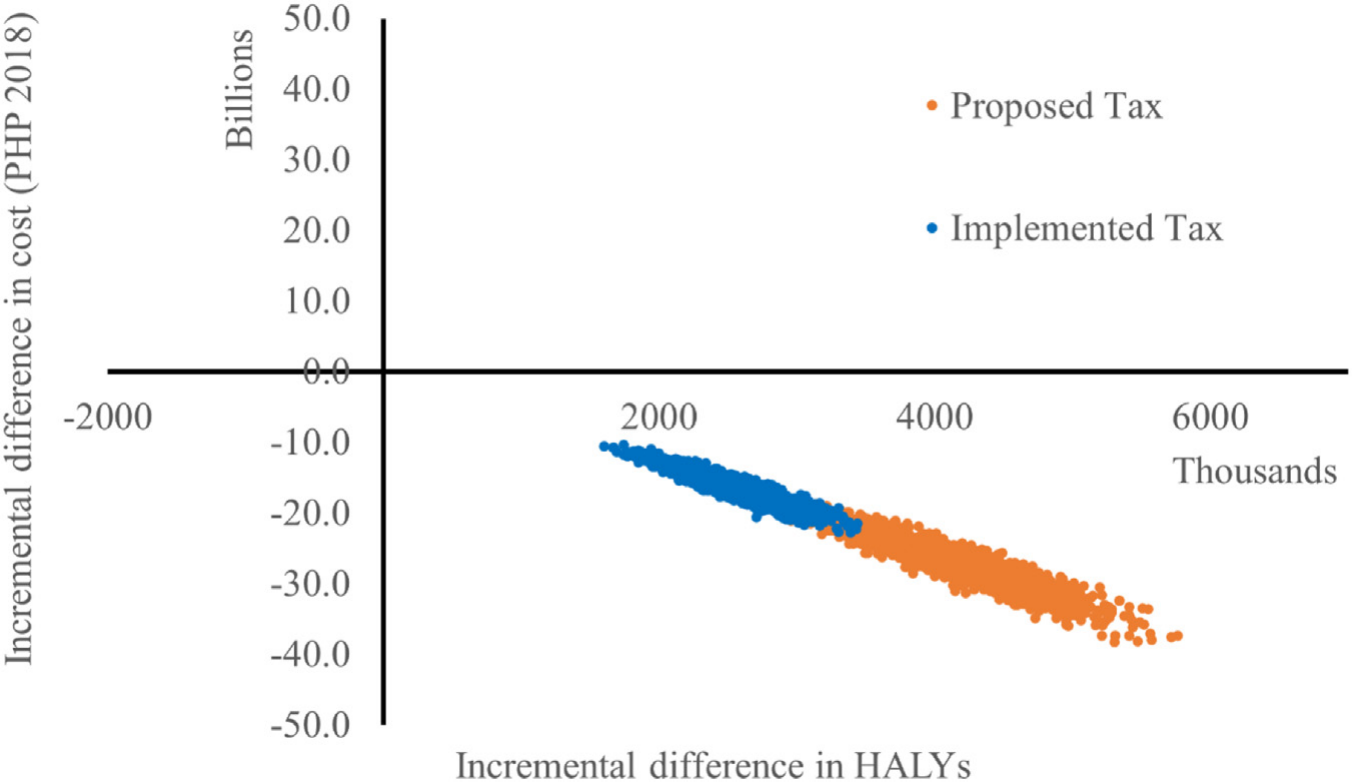
**Philippine SB tax cost-effectiveness plane**.

Table 4 shows the estimated health gain (quantified as HALYs) due to reduced mortality from overweight and obesity-related diseases, healthcare cost savings, and government taxation revenue, resulting from the implemented SB tax and the proposed SB tax in the Philippines, as well as the estimated policy implementation and monitoring costs. Fig. 2 shows the 2000 runs of the model for the implemented SB tax and the proposed SB tax plotted on a cost-effectiveness plane.

**Table 4:** Cost-utility results.

Parameter	Implemented SB tax	Proposed SB tax
Total incremental Health-adjusted Life Years (HALYs) gained	2,503,118 (95% UI: 1,947,500–3,097,076)	4,149,030 (95% UI: 3,278,074–5,053,183)
Total intervention costs^a^	PHP302M (95% UI: PHP198M–PHP410M)	PHP302M (95% UI: PHP198M–PHP410M)
Total healthcare cost offsets^a^	−PHP16.4B (95% UI: −PHP20.6B to −PHP12.6B)	−PHP27.3B (95% UI: −PHP33.9B to −PHP21.3B)
Total net costs^a^	−PHP16.1B (95%UI: −PHP20.3B to −PHP12.3B)	−PHP27.0B (95% UI: −PHP33.6B to −PHP21.0B)
Incremental cost-effectiveness ratio	Dominant (95% UI: Dominant–Dominant)	Dominant (95% UI: Dominant–Dominant)
Annual taxation revenue^a^	PHP12.7B (95% UI: PHP12.5B–PHP13.0B)	PHP17.6B (95% UI: PHP17.0B–PHP18.2B)
Total taxation revenue^a^	PHP426.3B (95% UI: PHP416.7B–PHP436.0B)	PHP589.4B (95% UI: PHP569.0B–PHP609.6B)

a All costs in PHP 2018 values; Negative costs equate to cost savings; All ‘total’ values presented for the lifetime of the population.

The proposed SB tax resulted in 4,149,030 (95% UI: 3,278,074–5,053,183 HALYs gained. The implemented SB tax was predicted to result in 39.7% fewer HALYs (HALYs gained: 2,503,118 (95% UI: 1,947,500–3,097,076)). Both the proposed and implemented SB tax scenarios were estimated to cost approximately PHP 302 million (M) (approximately USD 5.95M in 2018) (95% UI: PHP 198M–PHP410M) to implement.

Total healthcare cost savings resulting from the proposed SB tax were estimated at PHP 27.3 billion (B) (approximately USD 537M in 2018) (95% UI: PHP 21.3B; PHP 33.9B). This decreased by 39.9% to PHP 16.4 B (approximately USD 323M in 2018) healthcare savings under the implemented SB tax (95% UI: PHP 12.6B; PHP 20.6B).

Subsequently, the net costs of the proposed tax were estimated at -PHP 27.0B (approximately USD 532M in 2018) (95% UI: −PHP 33.6B; −PHP 21.0B) and the net costs of the implemented SB tax were estimated at −PHP 16.1B (approximately USD 317M in 2018) (95% UI: −PHP 20.3B; −PHP 12.3B). The ICERs for both the implemented and proposed SB tax scenarios were dominant (95% UI: dominant; dominant).

Annual government revenue resulting from the proposed SB tax was estimated at PHP17.6B (approximately US$345M in 2018) (95% UI: PHP17.0B; PHP18.2B) and total government revenue over the lifetime of the modelled population was estimated to be PHP589.4B (approximately US$11.6B in 2018) (95% UI: PHP569.0B; PHP609.6B). Conversely, annual government taxation revenue resulting from the implemented SB tax was 27.8% lower at PHP12.7B (approximately US$25M in 2018) (95% UI: PHP12.5B; PHP13.0B), while total government revenue over the lifetime of the modelled population was 27.7% lower at PHP426.3B (approximately US$8.4B in 2018) (95% UI: PHP416.7B−PHP436.0B).

### Sensitivity analysis

Supplementary Tables S4A–G show the results from various sensitivity analyses that were conducted.

Both Scenario A (coffee-based beverages included, tax rate 6.00 PHP) and Scenario B (coffee-based beverages excluded, tax rate 10.00 PHP) resulted in greater health gains and healthcare cost savings relative to the implemented SB tax, but slightly less gains relative to the proposed SB tax, as hypothesised. Scenario C (milk- and coffee-based beverages included, tax rate 10.00 PHP) resulted in greater health gains and healthcare cost savings relative to the initially proposed SB tax. In all cases the policy remained dominant (95% UI: dominant; dominant).

Both the proposed and the implemented SB taxes remained dominant (95% UI: dominant; dominant) when differing price elasticities were used^72^ (Scenario D). Further, when modelling a scenario where all healthcare savings per case of disease were reduced by 50% (Scenario E), and a scenario where policy costs were doubled (Scenario F), we found that the policy was still dominant (95% UI: dominant; dominant). Finally, the intervention remained dominant when modelling a scenario where childhood obesity weights were drawn from a systematic review.^73^

## Discussion

Herein, we present the first comparative modelling of proposed and implemented policies for taxing unhealthy beverages. Our findings suggest that the change in the Philippine SB tax design contributed to more than 1,600,000 HALYs worth of unrealised health benefits over the lifetime of the 2018 Philippine population. The subsequent additional government revenue was estimated to be almost 40% greater under the proposed SB tax.

Our modelling results suggest that the tax on SBs in the Philippines is likely to result in significant improvements to health, reductions in healthcare costs, and increases in government revenue in this country. The benefits of the SB tax in the Philippines have been previously modelled,^13^^,^^41^ though by incorporating policy implementation and monitoring costs our study represents the first full economic evaluation of the Philippine SB tax. Other studies support our findings that the tax is likely to have resulted in significant reductions in SB consumption^41^ as well as significant reductions in the burden of disease, reduced healthcare expenditure over time, and increased government revenue.^13^ The decline in SB consumption estimated herein is comparable to that estimated by Candy Hong Yi, Jing Wei, and Vaishnavi.^41^ Our study notably estimates that the Philippine SB tax has resulted in fewer healthcare cost savings when compared to Saxena et al.^13^ This is likely explained by Saxena et al.^13^'s use of Philippine Health Insurance Corporation (PhilHealth) data^74^ to estimate disease healthcare costs, which assumes that all cases of disease result in hospitalization—this is unlikely to be the case. Our approach accounts for the fact that many disease cases will not require hospitalization. Our estimate for government taxation revenue is also lower than that suggested by Saxena et al.^13^ This is likely due to our more conservative approach to estimating the impacts of the SB tax policy. While we did not model the impact of the tax across differing income groups, Saxena et al. estimate that lower income groups would bear the smallest tax burden.^13^ Candy Hong Yi, Jing Wei, and Vaishnavi^41^ also estimated that low income and rural populations would see the largest reduction in SB consumption in response to the tax, and so would likely see the greatest health benefit.

The potential benefits of SB taxation policies have been modelled in other countries in Southeast Asia: Indonesia,^48^ Thailand^23^ and Viet Nam.^75^ In all three countries, the implementation of a SB tax was estimated to reduce beverage consumption, disease cases, and healthcare expenditure.^23^^,^^48^^,^^75^ In both Thailand^23^ and Viet Nam,^75^ differing tax rates were modelled to determine the potential benefits from higher tax rates, though this was not done from the perspective of industry influence or tax policy changes. As was the case here, the authors found that higher tax rates would result in significantly greater benefits.^23^^,^^75^ Unlike the Philippines, the policies modelled in Indonesia,^48^ Thailand^23^ and Viet Nam^75^ all included coffee as a targeted beverage. This is significant as coffee-based beverages represent one of the most commonly consumed pre-packaged SBs in the Philippines, and the inclusion of these beverages in a SB tax scenario is likely to have significant implications for health and economic outcomes, as was the case herein.^39^^,^^40^

Although it is unknown whether food and beverage industry lobbying resulted in changes to the SB tax, literature strongly supports the assumption that the tax was changed in response to industry influence. Huse et al.^37^ described the influence of the food and beverage industry over policy processes in the Philippines and include descriptions made by policy makers of instances where industry influence had resulted in changes to policies, including the SB tax. This is further supported by Onagan, Ho, and Chua^12^ who highlight how industry lobbying resulted in specific changes to the SB taxation policy. This kind of industry influence over food policy design in the Philippines has also been described for policies related to school food environments and the marketing for breastmilk substitutes (BMS).^76^^,^^77^ Ultra-processed beverage corporations, such as The Coca-Cola Company, have identified the Philippines as a key growth market^78^ and so it is likely that such corporations may act to prevent the implementation of barriers to the sale of their products. Industry influence over food and beverage taxation policies is notable as elements of policy design, including tax rate, targeted nutrients or products, and the tax base (i.e., *ad valorem* or specific), can have significant impacts on policy outcomes.^79^ The results presented herein suggest that this documented lobbying by the food and beverage industry has the potential to impact health and economic outcomes from food and nutrition policies, notably through the lower tax rate and the exclusion of coffee-based beverages, a popular product in the Philippines.^39^^,^^40^

This analysis is the first to provide estimates of the potential health and economic impact of lobbying by the food and beverage industry on SB tax policy outcomes in the Philippines. The strengths of this study include our use of a previously validated cost-effectiveness model,^51^ our inclusion of cross-price elasticities to estimate the impact of beverage substitution, and our adaption of policy implementation and cost estimates to ensure that this analysis represents a full cost-utility analysis. Further, the model was built using nationally representative beverage consumption and anthropometric measurements representing the 2018 Philippine population, which aligns with the year the tax was implemented.^44^

There are several limitations that should be considered when interpreting these results. First, limited data was available to represent the Philippine context in relation to cost of policy implementation (which were taken from a Southeast Asia estimate^60^), nutrient composition of beverages (estimates were taken from Australia^47^), price elasticities (estimates were taken from the Mexican context^46^), healthcare costs (adapted using AIHW data^64^), and utility weights quantifying the quality of life impact of overweight and obesity in childhood (estimates were taken from Australia^57^^,^^58^). The impact of using varied assumptions were tested in sensitivity analyses and showed that the policy remained cost-effective when differing estimates were used. Further evidence of the disutility associated with BMI status for specific populations is also required. Second, while we use healthcare costs from Australia and adapt them to the Philippine context, there are key differences in the provision and therefore the cost of healthcare in these countries.^80^ The proportion of out-of-pocket healthcare expenditure is much higher in the Philippines (13.8% vs. 45.0%) and as such the Philippine Government does not directly receive all the estimated healthcare cost savings. However, any reduction in healthcare costs represents an economic benefit, and we include sensitivity analyses to account for potential bias from this approach, finding that the policy would remain cost-effective even if the reduction in healthcare costs was halved. Third, we did not include potential deadweight losses associated with taxation in our model. Fourth, we have taken a whole of government perspective, rather than a societal perspective, to estimate the potential cost-utility of the Philippine SB tax. Accordingly, we do not include the costs to industry in our model, including costs of lobbying and compliance, and potential loss of profits, nor do we consider productivity impacts of the policy. The impact of the SB tax on consumer costs and surplus are also excluded. Fifth, while we include cross-price elasticities in our model, we only estimate the likely substitution to other beverages, and do not look at the potential for consumers to increase their consumption of unhealthy foods. Future research should consider the likely impact of taxing SBs on total dietary consumption. Sixth, we used Philippine dietary data from a self-report survey^44^ which may have resulted in energy intakes being underestimated in our analysis, as self-report dietary surveys have been shown to frequently underestimate actual intake.^81^^,^^82^ This would also likely result in an underestimation of health benefits. Seventh, we take the mean price of SBs in the Philippines as calculated by Saxena et al.^13^ A more accurate approach would have been to conduct in-store audits. Eighth, we rely heavily on the GBD study, which uses relative risks from international literature.^83^ The relative risk of a given disease in the Philippines context may be lower or higher than global averages.^84^ Ninth, this model uses the caloric impact of SB consumption to estimate health benefits from the policy. This does not account for the non-caloric impacts of sugar and artificial sweetener consumption. Future modelling of population nutrition policies should incorporate the quality of diets and impacts on health outcomes rather than simply the caloric impact of interventions on BMI. Finally, we do not consider the potential impact of product reformulation by industry on energy intake and subsequent population health outcomes. Future research should explore the potential impact of product reformulation by the beverage industry (particularly in response to tiered tax rates). Product reformulation in the United Kingdom, in response to a tiered tax rate, resulted in reduced sugar content of beverages,^85^ and so it is likely that the impacts of the Philippine SB tax on energy intake would be enhanced in response to any product reformulation.

The Philippine government has implemented a cost-effective SB tax that is likely to result in significant health and economic benefits. However, our research shows that, to realise additional benefits, the Philippine government should consider expanding the tax to the rate and targeted products that were originally proposed. To support the findings presented herein, Philippine health and finance authorities should consider conducting an impact assessment of the SB tax to obtain real-world evidence on consumption. Such data is likely to strengthen future policy amendments and proposals.^12^

Our analysis demonstrates that lobbying by the food and beverage industry has the potential to influence policy outcomes and highlights the importance of considering mechanisms for reducing this influence, especially when considering future SB tax amendments. The importance of managing conflicts of interest and increasing transparency in interactions between policy makers and private enterprises in the Philippines, especially when it comes to food and nutrition policies, has been previously reported.^37^ Further, existing frameworks identify increased transparency, management of conflicts of interest, monitoring of and education about corporate practices, and prohibition of interactions between policy makers and industry as key strategies for reducing the impact of industry over policy processes.^86^, ^87^, ^88^, ^89^, ^90^ The public health sector should also be supported with additional resources, opportunities for training, and protection from industry threats.^90^ The expansive power of the UPF industry has been identified as a key barrier to addressing the influence of this industry, and so should also be addressed.^91^^,^^92^

We have shown that a tax on SBs represents a cost-effective policy option for improving population health and increasing government revenue in lower-middle income countries in East Asia. However, the influence of the food and beverage industry over policy processes has the potential to lessen the potential benefits of population nutrition policies. Strong mechanisms to manage and reduce conflicts of interest are needed to ensure that implemented nutrition policies align most closely with international best practice recommendations.

## Contributors

OH, KB, and JA were responsible for designing the study and formulating the research question. OH, with support from PN and JA, was responsible for data collection, and building and running the economic model. OH was responsible for drafting the manuscript. All authors provided feedback on this draft and read and approved the final manuscript.

## Data sharing statement

No individual participant data was used during the completion of this study. All data used in the construction of the economic model is publicly available to all at the references cited in text. Data is available for any purpose or use.

## Declaration of interests

OH, GS and KB are part of a project funded by VicHealth and UNICEF East Asia and Pacific that aimed to develop a research agenda to support improvement in the healthiness of urban retail food environments in the East Asia–Pacific Region. KB is supported by a fellowship from the 10.13039/501100001030Heart Foundation of Australia (102611). GS is a recipient of a 10.13039/501100000925National Health and Medical Research Council (NHMRC) Emerging Leadership Fellowship (2021/GNT2008535). JA is supported by a Deakin University postdoctoral research fellowship and the NHMRC funded Centre of Research Excellence in Food Retail Environments for Health (RE-FRESH) (APP1152968). JA also receives unrelated funding from the WHO, NHMRC, Australian Prevention Partnership Centre, the Cancer Council Western Australia, and the National Medical Research Council, Singapore. AP is a recipient of a NHMRC Investigator Grant and receives unrelated funding from the Australian Research Council and the Medical Research Futures Fund. AP is also a Board Director for Western Health and VicHealth. All other authors have no relevant funding sources to declare. MG, JPD, and IAA are employed by the Food and Nutrition Research Institute (FNRI) within the Philippine Department of Science and Technology. The FNRI provided policy support for the development and passing of the sweetened beverage tax and may indirectly benefit from revenue generated by the tax, as allocated by the Philippines Department of Finance. AC was employed by the Disease Prevention and Control Bureau, Philippine Department of Health at the time of the development and implementation of the sugar and sweetened beverage (SSB) tax. The Department of Health recommended the adoption of the SSB tax prior to its passing and may indirectly benefit from revenue generated by the tax, as allocated by the Philippines Department of Finance. All other authors have no conflicts of interest to declare.
