# Supplementary material for: A comparative analysis of the cost-utility of the Philippine tax on sweetened beverages as proposed and as implemented
Source: Lancet Reg Health West Pac. 2023 Sep 27;41:100912. doi: 10.1016/j.lanwpc.2023.100912 (PMC10534259; doi:10.1016/j.lanwpc.2023.100912)
Supplement: Supplementary File [file mmc1.docx]

**A comparative analysis of the cost-effectiveness of the Philippines’ tax on sweetened beverages as proposed and as implemented**

*Supplementary files*

Contents

[Table S1: Model input variables, data sources, and parameters 2](#_Toc141091173)

[Table S2: Model input variables, data sources, and parameters 3](#_Toc141091174)

[Table S3: Philippines BMI profile 4](#_Toc141091175)

[Table S4A: Sensitivity analyses modelling a tax with the proposed tax rate targeting the products included in the implemented tax 5](#_Toc141091176)

[Table S4B: Sensitivity analyses modelling a tax with the implemented tax rate targeting the products included in the proposed tax 6](#_Toc141091177)

[Table S4C: Sensitivity analyses modelling a ‘best-case’ tax with the proposed tax rate targeting products included in the proposed tax and additional products not included in either tax scenario 7](#_Toc141091178)

[Table S4D: Sensitivity analyses modelling the implemented tax with the healthcare cost offsets halved to account for uncertainty in calculations 8](#_Toc141091179)

[Table S4E: Sensitivity analyses modelling the implemented tax with the policy costs doubled to account for uncertainty in calculations 9](#_Toc141091180)

[Table S4F: Sensitivity analyses modelling the implemented tax sourcing beverage price elasticities from Chile 10](#_Toc141091181)

# Table S1: Model input variables, data sources, and parameters

| **Indicator** | **Philippines** | **Mexico** |
| --- | --- | --- |
| Population size* | 115,559,009  (2022) | 127,504,125  (2022) |
| Population age distribution*  0-15  15-64  65+ | 30%  64%  5%  (2022) | 25%  67%  8%  (2022) |
| Life expectancy at birth* | 69  (2021) | 70  (2021) |
| GDP per capita* | USD 3,498.5  (2022) | USD 11,091.3  (2022) |
| Proportion of population living in rural areas* | 48%  (2022) | 81%  (2022) |
| Gini coefficient* | 40.7  (2021) | 45.4  (2020) |
| Pre-tax sugar-sweetened beverage consumption (servings per day)^+^  Females aged 25 years  Females aged 35 years  Females aged 45 years  Females aged 55 years  Females aged 65 years  Females aged 75 years  Females aged 90 years  Males aged 25 years  Males aged 35 years  Males aged 45 years  Males aged 55 years  Males aged 65 years  Males aged 75 years  Males aged 90 years | 0.74  0.56  0.40  0.32  0.26  0.24  0.22  0.82  0.62  0.43  0.35  0.29  0.26  0.24  (2010 modelled data) | 2.40  1.87  1.37  1.09  0.88  0.79  0.71  2.61  2.03  1.48  1.19  0.97 0.87  0.79  (2010 modelled data) |

** The World Bank. 2023. https://www.worldbank.org/en/home*

*^+^ Singh GM, Micha R, Khatibzadeh S, Shi P, Lim S, Andrews KG, Engell RE, Ezzati M, Mozaffarian D; Global Burden of Diseases Nutrition and Chronic Diseases Expert Group (NutriCoDE). Global, Regional, and National Consumption of Sugar-Sweetened Beverages, Fruit Juices, and Milk: A Systematic Assessment of Beverage Intake in 187 Countries. PLoS One. 2015 Aug 5;10(8):e0124845. doi: 10.1371/journal.pone.0124845.*

# Table S2: Model input variables, data sources, and parameters

| **Input parameter** | **Values** | **Distribution** | **Sources** | **Key assumptions** |
| --- | --- | --- | --- | --- |
| **Baseline population** | | | | |
| Philippines BMI profile | Supplementary File S2 | Normal | 2018 Philippines Expanded National Nutrition Survey ^(1)^. |  |
| Baseline disease prevalence, incidence, and fatality rates | Global Burden of Disease study ^(2)^ | - | Global Burden of Disease study ^(2)^. |  |
| **Change in mean beverage and energy intake** | | | | |
| Tax rate | Implemented tax: 13.3%  Proposed tax: 22.2% | - | Saxena et al ^(3)^. |  |
| Beverage price elasticities and cross price elasticities | Colchero et al ^(4)^. | Normal | Colchero et al ^(4)^. | Price elasticities from Mexico are applicable in the Philippines context. |
| Per capita beverage consumption in the Philippines | Supplementary File S2 | Pert* (±50%) | 2018 Philippines Expanded National Nutrition Survey ^(1)^. |  |
| Energy content per beverage type | Soft drinks: 174 kJ/100mL  Energy and sports drinks: 191 kJ/100mL  Juice and fruit drink: 184 kJ/100mL  Milk and grain-based beverages: 266 kJ/100mL  Coffee-based beverages: 260 kJ/100mL | Pert* (±50%) | Australian Food Composition Database ^(5)^ | Nutrient contents of Australian beverages are applicable in the Philippines context. |
| **Tax costs and cost savings** | | | | |
| Cost of tax implementation | USD5,400,016 per 10 million population, for the lifetime of the intervention.  Include human resource costs, consultation costs, training costs, and policy monitoring evaluation costs. | Pert* (±50%) | WHO-CHOICE ^(6)^. | The cost of implementing taxes on tobacco is similar to the cost of implementing a tax on sweetened beverages.  Regional costs for Southeast Asia reflect potential costs in the Philippines. |
| Hospitalisation costs of relevant diseases | Australian Institute of Health and Welfare ^(7)^.  ‘Prevention and control of noncommunicable diseases in the Philippines: The case for investment’ ^(8)^. | - | Australian Institute of Health and Welfare ^(7)^.  ‘Prevention and control of noncommunicable diseases in the Philippines: The case for investment’ ^(8)^. | The proportionate cost of managing each included disease for one year is comparable between Australia and the Philippines. |
| Government tax revenue | Implemented tax: 6 PHP per litre  Proposed tax: 10 PHP per litre | - | Saxena et al ^(3)^. |  |
| ** The Pert distribution was used due to lack of available data on the distribution for these variables ^(9)^* | | | | |

# Table S3: Philippines BMI profile

| **Males** | | | **Females** | | |
| --- | --- | --- | --- | --- | --- |
| **Age** | **Mean BMI** | **SD** | **Age** | **Mean BMI** | **SD** |
| 2 | 15.85 | 3.75 | 2 | 15.69 | 2.60 |
| 3 | 15.35 | 1.83 | 3 | 15.33 | 2.01 |
| 4 | 15.03 | 2.27 | 4 | 15.00 | 1.80 |
| 5 | 14.96 | 2.27 | 5 | 14.91 | 1.80 |
| 6 | 15.01 | 2.27 | 6 | 14.80 | 1.80 |
| 7 | 15.13 | 2.27 | 7 | 14.82 | 1.80 |
| 8 | 15.50 | 2.27 | 8 | 15.22 | 1.80 |
| 9 | 15.89 | 2.27 | 9 | 15.65 | 1.80 |
| 10 | 16.20 | 2.27 | 10 | 16.15 | 1.80 |
| 11 | 16.73 | 2.27 | 11 | 16.66 | 1.80 |
| 12 | 17.09 | 3.16 | 12 | 17.77 | 2.97 |
| 13 | 17.70 | 3.11 | 13 | 18.51 | 3.09 |
| 14 | 18.16 | 2.81 | 14 | 19.24 | 3.05 |
| 15 | 18.91 | 2.99 | 15 | 19.69 | 2.95 |
| 16 | 19.46 | 3.09 | 16 | 19.98 | 3.19 |
| 17 | 19.98 | 3.05 | 17 | 20.31 | 3.30 |
| 18 | 20.70 | 3.40 | 18 | 20.53 | 3.29 |
| 19 | 20.97 | 3.45 | 19 | 20.90 | 3.67 |
| 20-24 | 22.24 | 3.60 | 20-24 | 22.47 | 3.92 |
| 25-29 | 23.19 | 3.71 | 25-29 | 23.73 | 4.18 |
| 30-34 | 23.57 | 3.76 | 30-34 | 24.37 | 4.26 |
| 35-39 | 24.00 | 3.86 | 35-39 | 25.13 | 4.34 |
| 40-44 | 24.11 | 3.82 | 40-44 | 25.27 | 4.41 |
| 45-49 | 24.13 | 3.91 | 45-49 | 25.21 | 4.33 |
| 50-54 | 23.68 | 3.87 | 50-54 | 24.98 | 4.38 |
| 55-59 | 23.23 | 3.88 | 55-59 | 24.66 | 4.53 |
| 60-64 | 22.88 | 3.82 | 60-64 | 24.17 | 4.45 |
| 65-69 | 22.31 | 3.76 | 65-69 | 23.70 | 4.54 |
| 70-74 | 22.00 | 3.54 | 70-74 | 23.03 | 4.39 |
| 75-79 | 21.38 | 3.37 | 75-79 | 22.30 | 4.21 |
| 80+ | 21.42 | 3.61 | 80+ | 21.42 | 4.18 |

# Table S4A: Sensitivity analyses modelling a tax with the proposed tax rate targeting the products included in the implemented tax

| **Parameter** | **Implemented SB tax** |
| --- | --- |
| ***Change in energy intake and weight*** | |
| Weighted average change in volume of beverages consumed (ml per person per day)* | -85.1 (95%UI: -88.5; -81.6) |
| Weighted average change in energy intake (kJ per person per day)* | -131.4 (95%UI: -160.5; -104.3) |
| Weighted average change in weight (kg per person per day)* | -1.22 (95%UI: -1.50; -0.97) |
| Weighted average change in BMI (kg/m^2^ per person per day)* | -0.59 (95%UI: -0.71; -0.47) |
| ***Cost effectiveness results*** | |
| Total HALYs gained | 4,112,712  (95%UI: 3,207,634; 5,079,794) |
| Total intervention costs^+^ | PHP300.9M  (95%UI: PHP198.3M; 407.0M) |
| Total healthcare cost offsets^+^ | -PHP27.0B  (95%UI: -PHP33.6B to -PHP20.7B) |
| Total cost savings^+^ | -PHP26.7B  (95%UI: -PHP33.3B to -PHP20.4B) |
| Annual taxation revenue | PHP18.4B  (95%UI: PHP17.9B; PHP18.9B) |
| Total taxation revenue | PHP615.6B  (95%UI: PHP599.7B; PHP630.9B) |
| Incremental cost-effectiveness ratio | Dominant (95%UI: dominant; dominant) |
| ** Weighted to the age and sex distribution of the 2018 Philippines population*  *^ Weight related diseases include cancers (colorectal, breast, endometrial, kidney), cardiovascular diseases ischaemic heart disease, hypertensive heart disease, ischaemic stroke), type 2 diabetes mellitus, and osteoarthritis (hip, knee)*  *^+^All costs in PHP2018 values; Negative costs equate to cost savings* | |

# Table S4B: Sensitivity analyses modelling a tax with the implemented tax rate targeting the products included in the proposed tax

| **Parameter** | **Implemented SB tax** |
| --- | --- |
| ***Change in energy intake and weight*** | |
| Weighted average change in volume of beverages consumed (ml per person per day)* | -53.8 (95%UI: -55.9; -51.7) |
| Weighted average change in energy intake (kJ per person per day)* | -85.9 (95%UI: -103.1; -68.8) |
| Weighted average change in weight (kg per person per day)* | -0.80 (95%UI: -0.97; -0.60) |
| Weighted average change in BMI (kg/m^2^ per person per day)* | -0.37 (95%UI: -0.44; -0.30) |
| ***Cost effectiveness results*** | |
| Total HALYs gained | 2,527,471  (95%UI: 2,962,899; 3,155,469) |
| Total intervention costs^+^ | PHP303.6M  (95%UI: PHP196.7M; 411.6M) |
| Total healthcare cost offsets^+^ | -PHP16.6B  (95%UI: -PHP21.0B to -PHP12.7B) |
| Total cost savings^+^ | -PHP16.3B  (95%UI: -PHP20.7B to -PHP12.4B) |
| Annual taxation revenue | PHP12.6B  (95%UI: PHP12.3B; PHP12.9B) |
| Total taxation revenue | PHP421.4B  (95%UI: PHP412.2B; PHP430.8B) |
| Incremental cost-effectiveness ratio | Dominant (95%UI: dominant; dominant) |
| ** Weighted to the age and sex distribution of the 2018 Philippines population*  *^ Weight related diseases include cancers (colorectal, breast, endometrial, kidney), cardiovascular diseases ischaemic heart disease, hypertensive heart disease, ischaemic stroke), type 2 diabetes mellitus, and osteoarthritis (hip, knee)*  *^+^All costs in PHP2018 values; Negative costs equate to cost savings* | |

# Table S4C: Sensitivity analyses modelling a ‘best-case’ tax with the proposed tax rate targeting products included in the proposed tax and additional products not included in either tax scenario

| **Parameter** | **Implemented SB tax** |
| --- | --- |
| ***Change in energy intake and weight*** | |
| Weighted average change in volume of beverages consumed (ml per person per day)* | -91.1 (95%UI: -94.5; -87.7) |
| Weighted average change in energy intake (kJ per person per day)* | -147.6 (95%UI: -176.4; -119.0) |
| Weighted average change in weight (kg per person per day)* | -1.37 (95%UI: -1.64; -1.10) |
| Weighted average change in BMI (kg/m^2^ per person per day)* | -0.63 (95%UI: -0.76; -0.51) |
| ***Cost effectiveness results*** | |
| Total HALYs gained | 4,439,662  (95%UI: 3,513,725; 5,469,454) |
| Total intervention costs^+^ | PHP301.1M  (95%UI: PHP193.7M; 407.3M) |
| Total healthcare cost offsets^+^ | -PHP29.5B  (95%UI: -PHP36.8B to -PHP22.8B) |
| Total cost savings^+^ | -PHP29.2B  (95%UI: -PHP36.6B to -PHP22.5B) |
| Annual taxation revenue | PHP17.9B  (95%UI: PHP17.4B; PHP18.4B) |
| Total taxation revenue | PHP597.8B  (95%UI: PHP582.7B; PHP614.0B) |
| Incremental cost-effectiveness ratio | Dominant (95%UI: dominant; dominant) |
| ** Weighted to the age and sex distribution of the 2018 Philippines population*  *^ Weight related diseases include cancers (colorectal, breast, endometrial, kidney), cardiovascular diseases ischaemic heart disease, hypertensive heart disease, ischaemic stroke), type 2 diabetes mellitus, and osteoarthritis (hip, knee)*  *^+^All costs in PHP2018 values; Negative costs equate to cost savings* | |

# Table S4D: Sensitivity analyses modelling the implemented tax with the healthcare cost offsets halved to account for uncertainty in calculations

| **Parameter** | **Implemented SB tax** |
| --- | --- |
| ***Change in energy intake and weight*** | |
| Weighted average change in volume of beverages consumed (ml per person per day)* | -51.1 (95%UI: -53.3; -49.0) |
| Weighted average change in energy intake (kJ per person per day)* | -78.6 (95%UI: -95.0; -62.0) |
| Weighted average change in weight (kg per person per day)* | -0.73 (95%UI: -0.89; -0.58) |
| Weighted average change in BMI (kg/m^2^ per person per day)* | -0.35 (95%UI: -0.42; -0.28) |
| ***Cost effectiveness results*** | |
| Total HALYs gained | 2,516,341  (95%UI: 1,960,567; 3,092,523) |
| Total intervention costs^+^ | PHP302.4M  (95%UI: PHP195.4M; 413.5M) |
| Total healthcare cost offsets^+^ | -PHP8.2B  (95%UI: -PHP10.3B to -PHP6.3B) |
| Total cost savings^+^ | -PHP7.9B  (95%UI: -PHP9.9B to -PHP6.0B) |
| Annual taxation revenue | PHP12.8B  (95%UI: PHP12.5B; PHP13.0B) |
| Total taxation revenue | PHP426.3B  (95%UI: PHP416.8B; PHP436.2B) |
| Incremental cost-effectiveness ratio | Dominant (95%UI: dominant; dominant) |
| ** Weighted to the age and sex distribution of the 2018 Philippines population*  *^ Weight related diseases include cancers (colorectal, breast, endometrial, kidney), cardiovascular diseases ischaemic heart disease, hypertensive heart disease, ischaemic stroke), type 2 diabetes mellitus, and osteoarthritis (hip, knee)*  *^+^All costs in PHP2018 values; Negative costs equate to cost savings* | |

# Table S4E: Sensitivity analyses modelling the implemented tax with the policy costs doubled to account for uncertainty in calculations

| ***Parameter*** | ***Implemented SB tax*** |
| --- | --- |
| ***Change in energy intake and weight*** | |
| Weighted average change in volume of beverages consumed (ml per person per day)* | -51.1 (95%UI: -53.2; -48.9) |
| Weighted average change in energy intake (kJ per person per day)* | -78.6 (95%UI: -95.8; -61.6) |
| Weighted average change in weight (kg per person per day)* | -0.73 (95%UI: -0.88; -0.57) |
| Weighted average change in BMI (kg/m^2^ per person per day)* | -0.35 (95%UI: -0.42; -0.28) |
| ***Cost effectiveness results*** | |
| Total HALYs gained | 2,506,678  (95%UI: 1,965,934; 3,121,476) |
| Total intervention costs^+^ | PHP608.5M  (95%UI: PHP397.0M; 826.9M) |
| Total healthcare cost offsets^+^ | -PHP16.5B  (95%UI: -PHP20.7B to -PHP12.6B) |
| Total cost savings^+^ | -PHP15.9B  (95%UI: -PHP20.1B to -PHP12.0B) |
| Annual taxation revenue | PHP12.8B  (95%UI: PHP12.5B; PHP13.0B) |
| Total taxation revenue | PHP426.3B  (95%UI: PHP416.7B; PHP436.0B) |
| Incremental cost-effectiveness ratio | Dominant (95%UI: dominant; dominant) |
| ** Weighted to the age and sex distribution of the 2018 Philippines population*  *^ Weight related diseases include cancers (colorectal, breast, endometrial, kidney), cardiovascular diseases ischaemic heart disease, hypertensive heart disease, ischaemic stroke), type 2 diabetes mellitus, and osteoarthritis (hip, knee)*  *^+^All costs in PHP2018 values; Negative costs equate to cost savings* | |

# Table S4F: Sensitivity analyses modelling the implemented tax sourcing beverage price elasticities from Chile

| ***Parameter*** | ***Implemented SB tax*** |
| --- | --- |
| ***Change in energy intake and weight*** | |
| Weighted average change in volume of beverages consumed (ml per person per day)* | -53.6  (85%UI: -57.6 to -49.8) |
| Weighted average change in energy intake (kJ per person per day)* | -82.9  (85%UI: -101.0 to -65.0) |
| Weighted average change in weight (kg per person per day)* | -0.77  (85%UI: -0.94 to -0.61) |
| Weighted average change in BMI (kg/m^2^ per person per day)* | -0.37  (85%UI: -0.45 to -0.30) |
| ***Cost effectiveness results*** | |
| Total HALYs gained | 4,282,817  (95%UI: 3,314,617; 5,334,238) |
| Total intervention costs^+^ | PHP302.6M  (95%UI: PHP194.3M; 410.2M) |
| Total healthcare cost offsets^+^ | -PHP28.1B  (95%UI: -PHP35.0B to -PHP21.6B) |
| Total cost savings^+^ | -PHP27.8B  (95%UI: -PHP34.7B to -PHP21.2B) |
| Annual taxation revenue | PHP12.6B (95%UI: PHP12.3B to PHP12.9B) |
| Total taxation revenue | PHP421.7B (95%UI: PHP411.3B to PHP432.1B) |
| Incremental cost-effectiveness ratio | Dominant (95%UI: dominant; dominant) |
| ** Weighted to the age and sex distribution of the 2018 Philippines population*  *^ Weight related diseases include cancers (colorectal, breast, endometrial, kidney), cardiovascular diseases ischaemic heart disease, hypertensive heart disease, ischaemic stroke), type 2 diabetes mellitus, and osteoarthritis (hip, knee)*  *^+^All costs in PHP2018 values; Negative costs equate to cost savings* | |

# Table S4G: Sensitivity analyses modelling the implemented tax sourcing childhood utility weights from a systematic review

| ***Parameter*** | ***Implemented SB tax*** |
| --- | --- |
| ***Change in energy intake and weight*** | |
| Weighted average change in volume of beverages consumed (ml per person per day)* | -51.0  (85%UI: -53.2 to -49.1) |
| Weighted average change in energy intake (kJ per person per day)* | -78.6  (85%UI: -95.8 to -62.1) |
| Weighted average change in weight (kg per person per day)* | -0.73  (85%UI: -0.89 to -0.58) |
| Weighted average change in BMI (kg/m^2^ per person per day)* | -0.35  (85%UI: -0.43 to -0.28) |
| ***Cost effectiveness results*** | |
| Total HALYs gained | 1,343,698  (95%UI: 1,029,746; 1,725,473) |
| Total intervention costs^+^ | PHP304.0M  (95%UI: PHP196.4M; 413.4M) |
| Total healthcare cost offsets^+^ | -PHP9.4B  (95%UI: -PHP12.1B to -PHP7.1B) |
| Total cost savings^+^ | -PHP9.1B  (95%UI: -PHP11.8B to -PHP6.7B) |
| Annual taxation revenue | PHP12.7B (95%UI: PHP12.4B to PHP13.0B) |
| Total taxation revenue | PHP426.1B (95%UI: PHP416.2B to PHP435.7B) |
| Incremental cost-effectiveness ratio | Dominant (95%UI: dominant; dominant) |
| ** Weighted to the age and sex distribution of the 2018 Philippines population*  *^ Weight related diseases include cancers (colorectal, breast, endometrial, kidney), cardiovascular diseases ischaemic heart disease, hypertensive heart disease, ischaemic stroke), type 2 diabetes mellitus, and osteoarthritis (hip, knee)*  *^+^All costs in PHP2018 values; Negative costs equate to cost savings* | |

References

1. Food and Nutrition Research Institute. eNutrition: The Philippines Knowledge Centre on Food and Nutrition: Republic of the Philippines; 2018 [Available from: <http://enutrition.fnri.dost.gov.ph/site/home.php>.

2. Salomon JA, Vos T, Hogan DR, et al. Common values in assessing health outcomes from disease and injury: disability weights measurement study for the Global Burden of Disease Study 2010. The Lancet. 2012;380(9859):2129-43.

3. Saxena A, Koon AD, Lagrada-Rombaua L, et al. Modelling the impact of a tax on sweetened beverages in the Philippines: an extended cost-effectiveness analysis. Bulletin of the World Health Organization. 2019;97(2):97-107.

4. Colchero MA, Salgado JC, Unar-Munguía M, et al. Price elasticity of the demand for sugar sweetened beverages and soft drinks in Mexico. Economics & Human Biology. 2015;19:129-37.

5. Food Standards Australia New Zealand. Australian Food Composition Database 2010 [Available from: <http://www.foodstandards.gov.au/science/monitoringnutrients/nutrientables/nuttab/Pages/default.aspx>.

6. Bertram MY, Chisholm D, Watts R, et al. Cost-Effectiveness of Population Level and Individual Level Interventions to Combat Non-communicable Disease in Eastern Sub-Saharan Africa and South East Asia: A WHO-CHOICE Analysis. Int J Health Policy Manag. 2021;10(11):724-33.

7. Nguyen P, Ananthapavan J, Tan EJ, et al. Modelling the potential health and economic benefits of reducing population sitting time in Australia. International Journal of Behavioral Nutrition and Physical Activity. 2022;19(1):28.

8. World Health Organization, United Nations Development Programme. Prevention and control of noncommunicable diseases in the Philippines: The case for investment. Geneva; 2019.

9. Barendregt JJ. Ersatz User Guide. Sunrise Beach, Queensland, Australia; 2017.
